# Intraspinal Grafting of Serotonergic Neurons Modifies Expression of Genes Important for Functional Recovery in Paraplegic Rats

**DOI:** 10.1155/2018/4232706

**Published:** 2018-07-25

**Authors:** Krzysztof Miazga, Hanna Fabczak, Ewa Joachimiak, Małgorzata Zawadzka, Łucja Krzemień-Ojak, Marek Bekisz, Anna Bejrowska, Larry M. Jordan, Urszula Sławińska

**Affiliations:** ^1^Nencki Institute of Experimental Biology, Polish Academy of Sciences, Warsaw, Poland; ^2^Department of Physiology and Phatophysiology, University of Manitoba, Winnipeg, MB, Canada

## Abstract

Serotonin (5-hydroxytryptamine; 5-HT) plays an important role in control of locomotion, partly through direct effects on motoneurons. Spinal cord complete transection (SCI) results in changes in 5-HT receptors on motoneurons that influence functional recovery. Activation of 5-HT_2A_ and 5-HT_7_ receptors improves locomotor hindlimb movements in paraplegic rats. Here, we analyzed the mRNA of 5-HT_2A_ and 5-HT_7_ receptors (encoded by *Htr2a* and *Htr7* genes, resp.) in motoneurons innervating tibialis anterior (TA) and gastrocnemius lateralis (GM) hindlimb muscles and the tail extensor caudae medialis (ECM) muscle in intact as well as spinal rats. Moreover, the effect of intraspinal grafting of serotonergic neurons on *Htr2a* and *Htr7* gene expression was examined to test the possibility that the graft origin 5-HT innervation in the spinal cord of paraplegic rats could reverse changes in gene expression induced by SCI. Our results indicate that SCI at the thoracic level leads to changes in *Htr2a* and *Htr7* gene expression, whereas transplantation of embryonic serotonergic neurons modifies these changes in motoneurons innervating hindlimb muscles but not those innervating tail muscles. This suggests that the upregulation of genes critical for locomotor recovery, resulting in limb motoneuron plasticity, might account for the improved locomotion in grafted animals.

## 1. Introduction

Motoneurons (MNs) respond to 5-HT with an increase in excitability [1–3]. We and others have previously argued that 5-HT_2A_ and 5-HT_7_ receptors are important in the initiation and control of locomotion [3–12], and that these receptors mediate hindlimb locomotor recovery produced in paraplegic animals after replacement of 5-HT neurons into the sublesional spinal cord by grafts of fetal brainstem [10, 13]. One of the effects of spinal cord transection, which interrupts the 5-HT pathway from the brainstem to the spinal cord, is plasticity in 5-HT receptors of spinal MNs [14, 15]. The 5-HT_7_ receptors have been implicated in control of MNs or reflexes involved in respiration, jaw movement, micturition, and locomotion [16–21] as well as in the control of pain after spinal cord injury [22, 23], while the 5-HT_2A_ receptor has been implicated in the control of respiration, development of spasticity in tail and hindlimb digit MNs, and the recovery of locomotor capability after spinal cord injury [24–27]. Intraspinal grafting of serotonergic neurons leads to functional recovery and involves activation of 5-HT_2A_ and 5-HT_7_ receptors [10]. We asked whether the facilitation of locomotion by our grafts might be mediated by plasticity in these key receptors that are necessary for locomotor recovery.

The 5-HT_7_ receptor protein is found in MNs of the spinal cord [28], with some MN populations (e.g., Onuf's nucleus) more intensely labeled than others. MNs in the L4 spinal cord, where MNs to hindlimb muscles are located, displayed a relatively low level of labeling. These receptors have been shown to have excitatory effects on some MNs, including phrenic MNs [29] and trigeminal MNs [19], but not hypoglossal respiratory MNs [30, 31].

The afterhyperpolarization (AHP) in many types of neurons is reduced by 5-HT, and this effect may be mediated by 5-HT_7_ receptors [19, 32]. MNs of limb muscles have reduced AHPs during locomotion [33, 34], and lamprey MNs have reduced AHP due to 5-HT [35, 36]. This effect serves as a means of increasing MN spiking.

The 5-HT_2A_ receptor is abundant in ventral horn MNs [37, 38], with variable expression levels depending upon the functional role of the cell. For example, 5-HT_2A_ receptors are differentially distributed on MNs to the physiological extensor soleus muscle and extensor digitorum longus, a physiological flexor muscle [39]. Plasticity in the 5-HT_2A_ receptor protein has been examined after sacral spinal cord injury, where the changes have been suggested to underlie the development of tail spasticity (reviewed in [14, 15]). Contusive spinal cord injury at the thoracic level resulted in upregulation of 5-HT_2A_ receptor protein in MNs of the rostral dorsolateral nucleus innervating the plantar muscles of the foot, with an associated increase in the H-reflex recorded from the plantar muscles of the hindpaw [40]. Cervical spinal cord hemisections give rise to increased 5-HT_2A_ receptor protein in phrenic MNs and their subsequent increased excitability [27].

Chopek et al. [41] demonstrated that the extensor monosynaptic reflex in hindlimb MNs of passively cycled spinal rats responded to quipazine (a 5-HT_2_ agonist). This plasticity could be related to changes in 5-HT receptors in MNs; 5-HT_2A_ receptor mRNA increased after injury and increased further after passive cycling [42]. An increase in 5-HT_2A_ mRNA after sacral SCI was observed in tail MNs [43]. Chopek et al. [42] found no change in 5-HT_7_ receptor gene expression in lumbar MNs 3 months after spinal cord transection, but passive cycling increased 5-HT_7_ receptor mRNA. Giroux et al. [44] found spinal 5-HT receptors increased at 15 and 30 days after spinal cord injury, but returned to baseline levels after 60 days or more. They used [3H]8-OH-DPAT to label 5-HT receptors, a ligand that can bind to 5-HT_7_ receptors.

There is increasing evidence that activation of specific serotonin receptors in the spinal cord is effective for enhancing locomotor recovery in spinal rats [11, 45, 46]. Out of many serotonergic receptors present in the spinal cord, the 5-HT_2A_ and 5-HT_7_ receptors are the major ones implicated in the control of locomotion [3, 6, 7, 12, 25, 47–49]. The most commonly used agonists that are effective in enhancing the locomotor hindlimb movements when applied systemically are quipazine, which has high affinity for both 5-HT_2A_ and 5-HT_2C_ receptors and 8-hydroxy-2-(di-n-propylamino)-tetralin (8-OH-DPAT), which binds selectively to 5-HT_7_ and 5-HT_1A_ receptors [46, 47, 50]. We previously demonstrated that intraspinal grafting of embryonic brainstem tissue containing serotonergic neurons below a thoracic total transection enhances recovery of hindlimb locomotor movements [10, 13, 51]. We also demonstrated that graft-related recovery is mediated in part by 5-HT_2A_ and 5-HT_7_ receptors because application of their antagonists diminished the restored hindlimb locomotor movements [10, 13]. Constitutive activity in 5-HT_2_ receptors is implicated in the recovery of locomotion after SCI [25, 52], and intrathecal application of a selective 5-HT_7_ receptor antagonist during unrestrained locomotion in intact adult rats [4] blocks voluntary locomotion. Similar effects were obtained with 5-HT_2_ antagonists [53].

Here, we hypothesize that the plastic changes in these receptors that occur after spinal cord injury might be affected by the restoration of 5-HT innervation from grafted 5-HT neurons so as to reverse or normalize these changes. This hypothesis is consistent with the findings that the presence of 5-HT or other ligands for these receptors can downregulate, upregulate, or desensitize these 5-HT receptors [54, 55]. We tested this hypothesis on identified MNs of the lumbar enlargement that innervate muscles with known actions during locomotion (ankle flexor (TA), extensor (GM), and tail elevator (ECM)), and we attempted to induce plasticity in the receptors on these MNs using thoracic spinal cord transection. We monitored the changes in receptor mRNA expression produced in these identified lumbar MNs at 1 month and 4 months after spinal cord transection, and we determined whether intraspinal grafting of embryonic serotonergic neurons in spinal rats reverses the effects of spinal total transection on expression of these genes. We show, for the first time, that intraspinal grafting of embryonic serotonergic neurons reverses injury-evoked changes in 5-HT_2A_ and 5-HT_7_ receptor gene expression in the MN populations supplying hindlimb but not tail muscles. We propose that these changes may account for the effects of the grafts on neural plasticity responsible for locomotor recovery achieved by intraspinal grafting of embryonic raphe nuclei in paraplegic rats. A preliminary report of these findings has been published [56].

## 2. Materials and Methods

Experiments were performed on WAG (Wistar Albino Glaxo) 3-month-old female rats (*n* = 35) at the time of spinal cord injury. All procedures were conducted with care to minimize pain and suffering of animals with the approval of the First Local Ethics Committee in Poland, according to the principles of experimental conditions and laboratory animal care of European Union and the Polish Law on Animal Protection.

### 2.1. Spinal Cord Transection

Complete spinal cord transection (SCI) was performed (*n* = 23) at the Th9/10 level under deep anesthesia (isoflurane: 5% to induce and then maintained with 2% in oxygen 0.2–0.3 l/min and Butomidor: 0.05 mg/kg b.w.) as previously described [10]. To prevent the possibility of axonal regrowth through the cavity of the lesion, 1-2 mm of spinal cord tissue was aspirated using a glass pipette. Then, the muscles and fascia overlying the paravertebral muscles were closed in layers using sterile sutures, and the skin was closed with stainless steel surgical clips. After surgery, the animals received a nonsteroidal anti-inflammatory and analgesic treatment (s.c., Tolfedine 4 mg/kg b.w.) and antibiotics (s.c., Baytril 5 mg/kg b.w.; gentamicin 2 mg/kg b.w.) for the following 5–7 days. The bladder was emptied manually twice a day until the voiding reflex was reestablished.

### 2.2. Grafting of Embryonic 5-HT Cells

One month after SCI, nine out of 23 spinal rats were selected randomly for intraspinal grafting of embryonic serotonergic cells (SCI_TR_). Fourteen-day-old embryos (E14; E0—the day after mating) from time-pregnant female WAG rats were removed by Caesarean section and transferred to Hanks' buffered solution containing 0.5% glucose. A small piece of the embryonic caudal brainstem area containing the B1, B2, and B3 serotonergic regions was dissected under a microscope (for more details, see [10, 57]).

At the same time, the spinal cord of a recipient rat (isoflurane anesthesia, 5% to induce and then maintained with 2% in oxygen 0.2–0.3 l/min) was exposed by a small laminectomy at the Th11/12 vertebrae level (at least one segment below the total spinal cord transection), and a solid piece of embryonic tissue (approximately 2 *μ*l) was injected by pressure into the spinal cord 1 mm below the pial surface through a sharpened micropipette attached to the Hamilton syringe. The micropipette was then slowly withdrawn to avoid graft movement. Control spinal rats (*n* = 11) were subjected to a sham grafting procedure where the operation was identical to that described above, but no tissue was injected into the spinal cord [10, 13, 51].

### 2.3. Behavioral Assessment of Locomotor Ability in Spinal Rats

Before starting the procedure of collecting data for qRT-PCR analysis, two months after grafting (3 months after complete spinal cord transection), all the rats were subjected to behavioral testing to confirm the quality of their hindlimb plantar stepping. This was established in rats suspended above a treadmill with their forelimbs and thorax placed on a platform and with their hindlimbs touching the treadmill belt. To elicit hindlimb movements, a tail pinch was used. Stimulation of tail has been used for eliciting locomotion in many cases of complete spinal cord transection [46, 58] and has been used in all prior attempts to reveal locomotor recovery after brainstem neuron grafting [10, 13, 57, 59, 60]. The tail stimulus was adjusted by the experimenter to maximize the quality of plantar stepping. All the spinal grafted rats considered for the further investigation of gene expression in defined MNs or for immunohistochemistry of the spinal cord presented good plantar walking performance and were not different from those described in our previous paper [10, 13, 51, 57].

### 2.4. Implantation of EMG Electrodes

To evaluate the quality of hindlimb movements, we routinely use electromyography (EMG). In rats from the SCI_4m_ group (three months after total transection) and in the rats from the SCI_TR_ group (two months after intraspinal grafting), bipolar electrodes for EMG recordings were implanted under isoflurane anesthesia (5% to induce and then maintained with 2% in oxygen 0.2–0.3 l/min) in Sol muscle (physiological extensor active during the stance phase of the step cycle) and TA muscle (physiological flexor active during the swing phase of the step cycle) of both hindlimbs. The electrodes were made of Teflon-coated stainless steel wire (0.24 mm in diameter; AS633, CoonerWire Co., Chatsworth, CA, USA). The tips of the electrodes with 1–1.5 mm of the insulation removed were pulled through a cutaneous incision on the back of the animal, and each of the hook electrodes was inserted into the appropriate muscle and secured by a suture [10, 51, 57]. The distance between the electrode tips in the muscle was 1-2 mm. The ground electrode was placed under the skin on the back of the animal in some distance from the hindlimb muscles. The connector with the other ends of the wires fixed to it, covered with dental cement (Spofa Dental, Prague, Czech Republic) and silicone (3140 RTV, Dow Corning), was secured to the back of the animal. After surgery, the animals received antibiotic treatment (Baytril, 5 mg/kg s.c.).

### 2.5. Tissue Preparation and Immunohistochemistry: Morphological Verification of Spinal Reactive Gliosis in Spinal Rats with and without the Graft

After testing the quality of locomotor hindlimb movements, three grafted rats and three spinal control rats without the graft were subjected to perfusion to prepare the spinal cord tissue for histological investigation of the graft condition and presence of inflammatory responses at the spinal level in which the MNs were collected.

For immunochemistry, the spinal cords were collected from the animals deeply anesthetized with pentobarbital and transcardially perfused with cold 0.1 M phosphate-buffered saline (PBS), pH 7.2, for 2-3 min and subsequently with cold 4% paraformaldehyde in PBS for 15 min. The spinal cords were postfixed and then cryoprotected gradually up to 30% sucrose in PBS, embedded in OCT, frozen on dry ice, and sectioned in a cryotome (12 *μ*m). The collected cross sections were immobilized on poly-L-lysine-covered glass slides and stored at −20°C.

The primary antibodies that were used in the immunohistochemistry were mouse antiglial fibrillary acidic protein (GFAP, 1 : 1000, BD Pharmingen) for astrocytes and mouse neuronal nuclear protein antibody (NeuN, 1 : 100 Millipore) for neuronal labeling. Isolectin B4 (FITC-conjugated, 20 *μ*g/ml, Sigma-Aldrich) was used for activated microglia detection. To identify 5-HT-positive fibers, rabbit anti-5-HT antibody (1 : 1000, ImmunoStar) was used. The Alexa Fluor secondary antibodies were used: 488-conjugated donkey antibody against mouse IgG, 647-conjugated goat antibody against mouse IgG, and 555-conjugated donkey antibody against rabbit IgG, (1 : 1000 Invitrogen). Nuclei were stained with DAPI dye (1 *μ*g/ml in H_2_O, Sigma-Aldrich).

For the immunostaining, frozen cross sections, after several rinses in PBS, were blocked in 10% normal donkey or donkey/goat serum with 0.5% Triton X-100 in PBS at RT for 2 h and then incubated overnight at 4°C with primary antibodies followed by incubation with fluorophore-conjugated secondary antibodies for 2 h at room temperature. The specimens were coverslipped in fluorescence mounting medium (Dako) after several washes in PBS and examined on either a Zeiss Fluorescence Microscope Axio Imager.M2 or a Leica SP5 Laser Scanning Confocal Microscope.

### 2.6. Motoneuron Labeling

**Figure 1:**
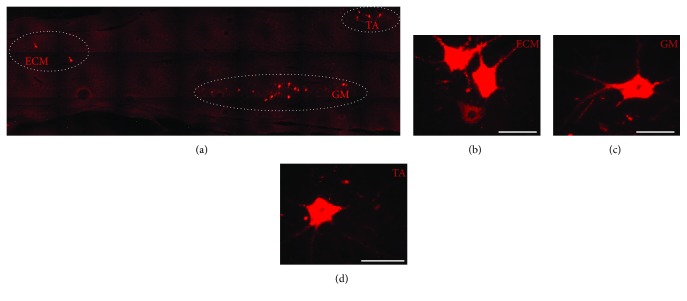
(a) Examples of labeled motoneurons (cholera toxin subunit B coupled with Alexa Fluor 555) innervating selected muscles: extensor caudae medialis (ECM) of the right side (b), gastrocnemius lateralis (GM) in the left hindlimb (c), and tibialis anterior (TA) in the right hindlimb (d). Scale bar: 50 *μ*m.

One week before the animal euthanasia, populations of MNs innervate the following muscles: TA of the right hindlimb, GM of the left hindlimb, and ECM of the right side of the tail were labeled by intramuscular injection by cholera toxin subunit B (CTB) coupled with Alexa Fluor 555 dye (see examples of CTB-labeled MNs innervating TA, GM, and ECM muscles in Figure 1). The TA and GM muscles were selected on the left and right sides to provide easy differentiation between the two MN pools innervating these two muscles when the same type of labeling was used in both of them. Also, the left ECM pool MN labeling (the most caudally localized) is a marker of the left side helping in identification of the GM MN pool. At the same time, the ECM and TA MN pools are separated with the most rostrocaudal distance. The ECM muscle was also selected because it plays a role in elevation of the tail, an indicator of functional recovery after spinal cord injury, according to the BBB score [61]. Animals were anesthetized using isoflurane (5% to induce and then maintained with 2% in oxygen 0.2–0.3 l/min), and proper muscles were exposed by small skin incisions enabling injection with 0.5% CTB using Hamilton syringe (TA and GM muscles 3 × 15 *μ*l of CTB and ECM muscle 2 × 20 *μ*l of CTB). The CTB injection was performed over a period of 2 minutes, the needle was slowly (2 min) withdrawn, and the skin incision was closed.

### 2.7. Htr2a and Htr7 Gene Expression Analysis

#### 2.7.1. Experiment Scheme

In order to examine the *Htr2a* and *Htr7* gene expression in MNs innervating selected hindlimb and tail muscles, we performed experiments on 29 WAG female rats, in which specific motoneuron pools were labeled by injection of retrograde tracer molecules into a target muscle. Our investigations were performed on rats from four experimental groups: INT: intact (*n* = 12); SCI_TR_: spinal cord injury followed by intraspinal transplantation of embryonic brainstem tissue containing serotonergic neurons (*n* = 6); SCI_1m_: 1 month after spinal cord injury (*n* = 5); SCI_4m_: 4 months after spinal cord injury (*n* = 6)-the time matched with the time of euthanasia of rats from the SCI_TR_ group.

Animals from the SCI_1m_ and SCI_4m_ groups were subjected to a spinal cord total transection procedure performed at the thoracic level. Animals from the INT group were the control animals that were not subjected to any procedure. Animals from the SCI_TR_ group received an intraspinal graft of 14-day-old rat embryonic brainstem one month after spinal cord total transection. Three months after intraspinal grafting in SCI_TR_ group and four months after complete transection in rats of both groups, SCI_TR_ and SCI_4m_ (seven days after motoneuron tracer injection with cholera toxin subunit B coupled with Alexa Fluor 555) rats were transcardially perfused with PBS solution to remove any blood-borne 5-HT receptors, and the spinal cord was dissected and prepared for further analysis (animals from the SCI_4m_ and SCI_TR_ groups were euthanized 4 months after spinal cord total transection).

#### 2.7.2. Laser Capture Microdissection (LCM) of Selected Motoneurons

Spinal cord tissue was collected from animals that were deeply anesthetized and transcardially perfused with PBS solution. Then, the spinal cords were immediately dissected and frozen by fast immersion in isopentane (−80°C) and stored at −80°C until future processing. Horizontal sections (20 *μ*m) of the lumbar-sacral spinal cord fragment were cut on the cryostat in −20°C and mounted on PEN Membrane Frame Slides (Applied Biosystems). Slides were stored at −80°C until future use.

Slides were dehydrated by immersion in increasing concentrations (70%, 90%, and 100%) of ethyl alcohol (ETOH) followed by immersion in xylene. Motoneuron collection was performed using an Arcturus Laser Microdissection System (Applied Biosystems). Tissue sections were photographed using a filter set for Alexa Fluor 555 to identify CTB-labeled MNs. Individual MNs were dissected using UV laser and collected on *Cap Sure Macro LCM Caps* (Applied Biosystems). After incubation in lysis buffer (*Arcturus Pico Pure RNA Isolation Kit*, Applied Biosystems) for 30 minutes, the samples were stored at −80°C until future processing.

#### 2.7.3. RNA Isolation

RNA was isolated using an *Arcturus Pico Pure RNA Isolation Kit* (Applied Biosystems) according to the manufacturer's instructions. Any residual genomic DNA was removed using supplementary DNAse I (*RQ1 RNase-Free DNase*, Promega) treatment. Three independent measurements of RNA concentration were obtained using NanoDrop (Thermo Scientific). RNA samples were pooled by mixing the same RNA amount isolated from one motoneuron population from all rats in a particular experimental group (i.e., equal amounts of RNA collected from the particular selected motoneuron populations in different rats in the same experimental group were pooled). RNA samples were stored at −80°C.

#### 2.7.4. Reverse Transcription PCR and cDNA Preamplification

Reverse transcription PCR (RT-PCR) was performed in a *MJ Mini Personal Thermal Cycler* (Bio-Rad) using a *High Capacity cDNA Reverse Transcription Kit* (Applied Biosystems) according to the manufacturer's instructions (10 min, 25°C; 120 min, 37°C; 5 min, 85°C; storage 4°C) with use of 31 ng of purified RNA as a sample. Synthesized cDNA was stored at −20°C for future use.

Preamplification was performed to increase the number of cDNA copies to the level necessary for accurate detection in the real-time PCR reaction. cDNA was preamplified in *MJ Mini Personal Thermal Cycler* (Bio-Rad) using *TaqMan PreAmp Master Mix Kit* (Applied Biosystems). Reactions were conducted in standard conditions according to the preamplification kit manufacturer's instructions: 95°C for 10 min; then 14 cycles: 95°C for 15 s; 60°C for 4 min.

#### 2.7.5. Real-Time PCR

Expression of the *Htr2a and Htr7* genes (encoding the 5-HT_2A_ and 5-HT_7_ receptors, resp.) in the selected MN populations was measured by semiquantitative real-time PCR (qRT-PCR) using a *Step One Plus* (Applied Biosystems) thermocycler and TaqMan gene-specific FAM/MGB assays (Applied Biosystems). *Ppia* gene encoding cyclophilin A was used as the housekeeping gene. The description of TaqMan assays used in the qRT-PCR reaction is shown in Table 1.

Reactions were run in triplicate for each sample and for each assay in 20 *μ*l reaction mix prepared in accordance with *TaqMan Gene Expression Master Mix* (Applied Biosystems) manufacturer recommendations. Reactions were run in standard, recommended by *TaqMan Gene Expression Master Mix* manufacturer conditions (2 min, 50°C; 10 min, 95°C; then 40 cycles: 15 s, 95°C; 1 min, 60°C). Relative expression levels of analyzed genes were then calculated using the comparative CT method.

### 2.8. Statistical Analysis

For comparison of results from two experimental groups, Student's *t*-test analysis was used after normal distribution was confirmed using Shapiro-Wilk test (Prism, GraphPad Software, La Jolla, CA). For statistical analysis of the results collected from qRT-PCR of more than two experimental groups that were expressed in relation to those established in INT rats, the nonparametric Kruskal-Wallis test for multiple independent sample comparison followed by Conover post hoc (further adjusted by the Holm family-wise error rate (FWER) method and in one case the method of Benjamini-Hochberg false discovery rate (FDR) when the FWER method just approached significance) was used (http://astatsa.com/KruskalWallisTest/).

## 3. Results

### 3.1. Locomotor Ability in Spinal Rats

**Figure 2:**
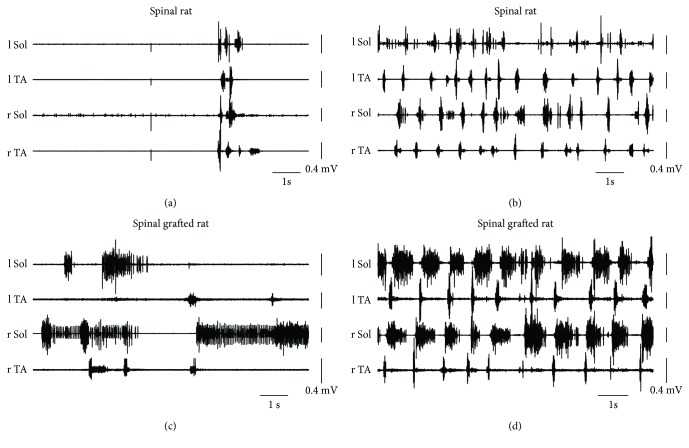
Examples of EMG recordings from hindlimb muscles of the spinal rats (a) and (b) and spinal grafted rat (c) and (d) during exploratory behavior in the home cage (a) and (c) and during locomotor-like hindlimb movements on a treadmill induced by tail pinching (b and d). Note that in spinal control rats during spontaneous movement in the home cage, the hindlimb muscles were usually silent but a brief episode of EMG activity could be induced by a sudden touch of the rat tail (a), while in the spinal grafted rats, the spontaneous EMG activity of Sol and TA muscles could be obtained without any external intervention (c) (for more details, see [10, 13, 51, 57]. Sol: soleus muscle; TA: tibialis anterior muscle; l/r: left/right.

As we published before, the SCI rats (SCI_1m_ and SCI_4m_) presented very limited hindlimb movements [10, 11, 13, 46, 51]. Usually, their hindlimbs were outstretched passively behind the hindquarters without any spontaneous hindlimb movement present and there was hardly any EMG activity recorded in the hindlimb flexor (TA) and extensor (Sol) muscles (Figure 2(a)). In contrast, the hindlimbs of the SCI_TR_ rats moving in the home cage were abducted with partial flexion in the ankle joint or flexed at all joints with the foot plantar surface touching the ground, but without noticeable body weight support in spite presence of some EMG activity in hindlimb Sol and TA muscles (Figure 2(c)). Tail pinching in SCI rats suspended over a treadmill induced only irregular, limited hindlimb movements characterized by obvious lack of body weight support with no plantar stepping. EMG activity of hindlimb flexor and extensor muscles was irregular with lack of the long burst of Sol EMG activity related to the stance phase of the step cycle that is observed in normal locomotor activity of intact rats (Figure 2(b)). In SCI_TR_ rats suspended over the treadmill, tail pinch was effective to induce regular plantar hindlimb walking with nice regular EMG muscle activity with typical alternating pattern of EMG Sol versus TA bursts of activity and long burst of Sol related to the stance phase of step cycle in both hindlimbs (Figure 2(d)).

### 3.2. Morphological Verification of Reactive Gliosis in Spinal Rats with and without the Graft

**Figure 3:**
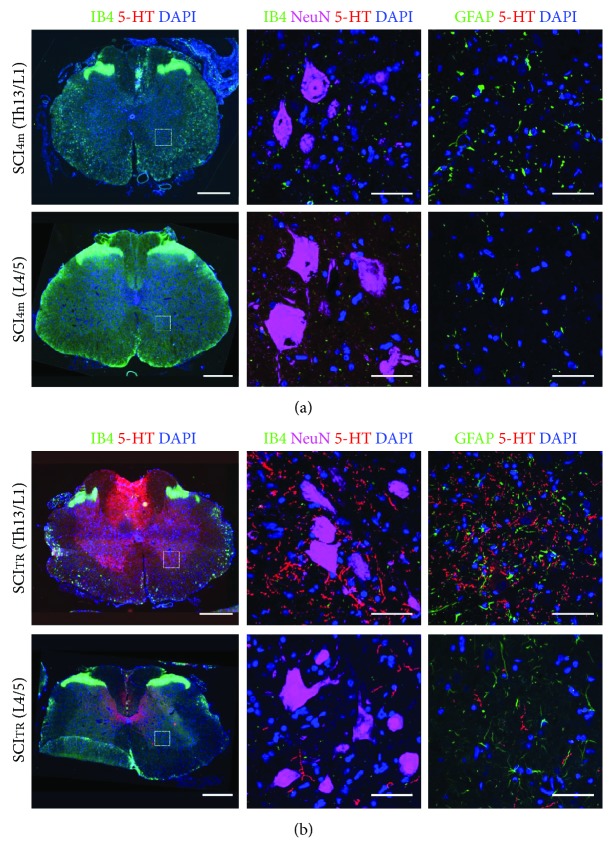
Immunofluorescent detection of inflammatory response in control SCI_4m_ (a) and transplanted SCI_TR_ (b) rats at 4 months after total spinal transection and 3 months after transplantation (sham grafting in SCI_4m_). The first panel shows general view of spinal cord cross section labeled with isolectin B4 (IB4) to visualize activated microglia in low power; the next two panels show boxed area labeled with IB4 (green; left and middle panel) for microglia and GFAP (green; right panel) for astrocytes, reimaged using confocal microscope. Note grafted tissue at Th13/L1 and serotonin-positive fibers (5-HT, red) located closely to ventral horn motoneurons (NeuN; purple) at L4/L5. Representative images: scale bar: 500 *μ*m and 50 *μ*m in low- and high-power images, respectively.

As we published before [10, 51, 57], intraspinally grafted 5-HT neurons survive the grafting procedure and their axons grow into the distal part of the host spinal cord, spreading caudally for a considerable distance below the total transection.  Figure 3(b) shows a representative example of fetal grafted tissue located in the spinal cord one/two segments below the total transection of the spinal grafted rat. The sham-operated spinal SCI_4m_ rats do not possess any 5-HT innervation in the spinal cord below the total transection (Figure 3(a)). Here, we also verified the presence of activated microglia and astrocytes in the spinal cord below transection in spinal rats with (SCI_TR_) and without the graft (SCI_4m_) long after the injury (4 months). While there was moderate microglia activation detectable within white matter and virtually no activation in grey matter at Th13/L1 (the level of a real or sham grafting), we were not able to detect any sign of inflammation at L4/L5 in any of the animals, regardless of whether they were sham or grafted ones (Figure 3 left and middle panels). Moreover, while we identified a slightly increased number of astrocytes in ventral horns at the grafting level compared to control, the same numbers of astrocytes were observed at L4/L5 in both SCI_4m_ and SCI_TR_ rats. However, none of the detected astrocytes showed the morphology of reactive cells (Figure 3, right panel). Thus, we assumed that 4 months after transplantation of embryonic tissue, there is no inflammatory reaction in the ventral horn at and below of L4/L5 spinal cord levels, where our samples were taken. This finding demonstrates that a role for inflammation in changes in 5-HT receptors such as previously suggested after sacral spinal cord injury [43] does not apply in our case.

### 3.3. Htr2a and Htr7 Gene Expression in MNs of Intact Adult Rats

**Figure 4:**
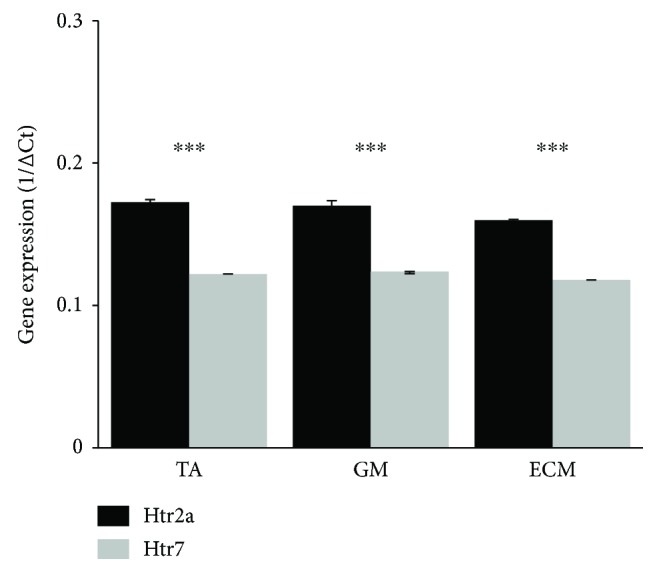
Expression level of *Htr2a* and *Htr7* genes (encoding 5-HT_2A_ and 5-HT_7_ receptors, resp.) in motoneuron populations innervating TA, GM, and ECM muscles in intact adult rats. Expression levels of *Htr2a* and *Htr7* genes were normalized to the expression level of the constitutively active *Ppia* (cyclophyline A) gene and was presented as the 1/ΔCt value of the analyzed genes. Student's *t*-test ^∗∗∗^*p* < 0.0001. Data are presented as mean **±** standard deviation (mean **±** SD). TA: tibialis anterior muscle; GM: gastrocnemius muscle; ECM: extensor caudae medialis muscle.

The relative abundance of the distinct mRNAs was established by the minimal number of amplification cycles necessary to detect a given mRNA and was presented at graph as 1/ΔCt value. In all analyzed populations of MNs innervating the TA, GM, and ECM muscles, expression of *Htr2a* and *Htr7* genes (encoding 5-HT_2A_ and 5-HT_7_ receptors, resp.) was detectable and expression of *Htr2a* gene in all analyzed motoneurons was significantly higher (Student's *t*-test *p* < 0.0001) than expression of gene encoding 5-HT_7_ serotonin receptor (Figure 4).

### 3.4. Htr2a Gene Expression in MNs

**Figure 5:**
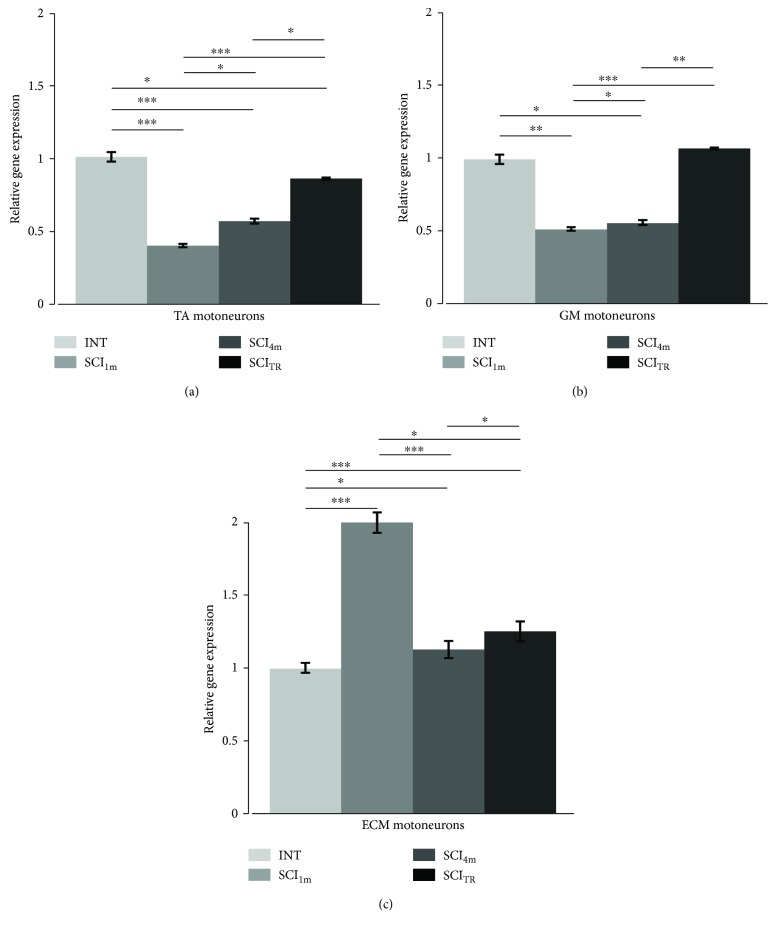
Changes in the *Htr2a* gene expression in MNs innervating the TA (a), GM (b), and ECM (c) muscles in rats one and four months after total spinal cord transection and in spinal rats with a graft of 14-day-old rat embryonic brainstem tissue containing serotonergic neurons. INT: intact rats; SCI_1m_: rats 1 month after spinal cord transection; SCI_4m_: rats 4 months after spinal cord transection; SCI_TR_: rats 4 months after spinal cord transection with a graft; TA: tibialis anterior muscle; GM: gastrocnemius muscle; ECM: extensor caudae medialis muscle. Data normalized to the expression of *Htr2a* gene in MNs of INT rats are presented as mean **±** SD (standard deviation). Nonparametric Kruskal-Wallis test with Conover post hoc method for multiple comparison (^∗^*p* < 0.05; ^∗∗^*p* < 0.01; ^∗∗∗^*p* < 0.001).

In comparison to INT rats, in SCI_1m_ rats, the *Htr2a* gene expression was reduced by ~60% in TA MNs (nonparametric Kruskal-Wallis test (df: 3, *p* = 0.015) with Conover post hoc test *p* < 0.001) and by ~50% in the GM MNs (nonparametric Kruskal-Wallis test (df: 3, *p* = 0.019) with Conover post hoc test *p* < 0.01) and increased by 103% in ECM MNs (nonparametric Kruskal-Wallis test (df: 3, *p* = 0.015) with Conover post hoc *p* < 0.0001) when normalized to the *Htr2a* gene expression in MNs of INT animals (Figure 5).

In SCI_4m_ rats, the *Htr2a* gene expression was also lower than in INT rats (~45% of INT) in TA MNs (Conover post hoc test *p* < 0.001) and GM MNs (Conover post hoc test *p* < 0.05). There was also a small difference in gene expression in SCI_4m_ rats in comparison to SCI_1m_ rats (Conover post hoc test *p* < 0.05) in MNs of both TA and GM muscles. In SCI_4m_, the *Htr2a* gene expression in the ECM MNs decreased by ~44% (Conover post hoc *p* < 0.001) in comparison to the gene expression level measured in SCI_1m_ rats. Expression of the *Htr2a* gene in TA and GM MNs of SCI_4m_ rats was still much lower than expression of this gene in INT rats (Figure 5).

Intraspinal grafting of embryonic brainstem serotonergic neurons reversed to some extent changes in the *Htr2a* gene expression observed in the TA and GM MNs of SCI_1m_ rats (Figure 5). Expression of the *Htr2a* gene in TA and in GM MNs in SCI_TR_ rats was significantly higher by 112% and 110%, respectively, than that in SCI_1m_ (Conover post hoc test *p* < 0.001 for both MN populations) and by 52% and 93%, respectively, than that in SCI_4m_ (Conover post hoc test *p* < 0.05 and *p* < 0.01, resp., for both MN populations). However, *Htr2a* gene expression remained ~14% lower in comparison to MNs of INT TA (Conover post hoc test *p* < 0.05) but in the GM MNs was restored to the level of INT (Conover post hoc test *p* > 0.05). The expression of the *Htr2a* gene in ECM MNs of SCI_TR_ rats remained increased by ~25% in comparison to that in MNs of INT rats (Conover post hoc test *p* < 0.001).

### 3.5. Htr7 Gene Expression in MNs

**Figure 6:**
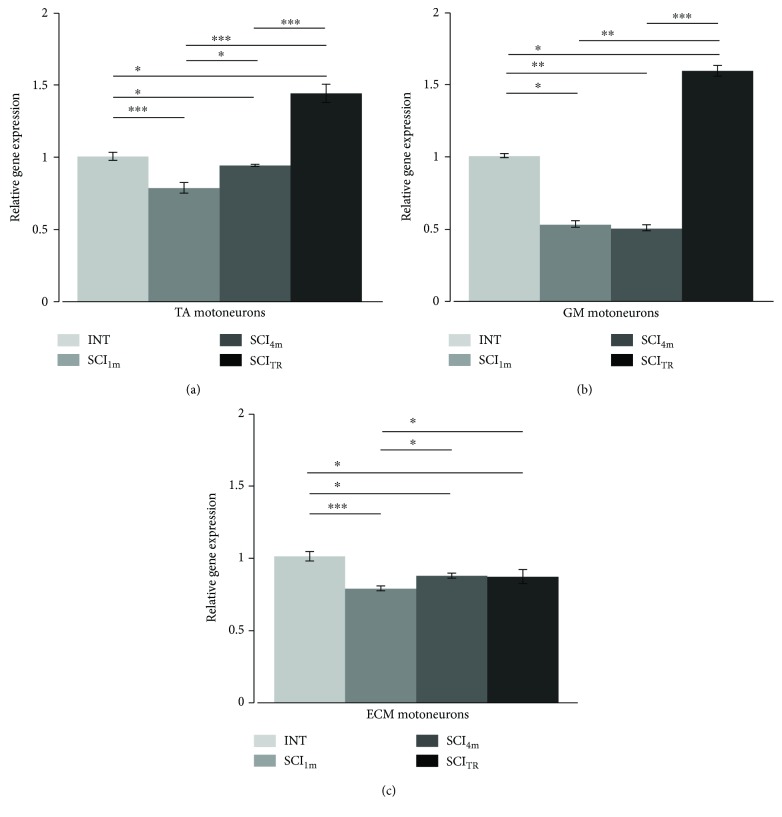
Changes in the *Htr7* gene expression in MNs innervating TA (a), GM (b), and ECM (c) muscles in rats one and four months after total spinal cord transection and in spinal rats with a graft of 14-day-old rat embryonic brainstem tissue containing serotonergic neurons. INT: intact rats; SCI_1m_: rats 1 month after spinal cord transection; SCI_4m_: rats 4 months after spinal cord transection; SCI_TR_: rats 4 months after spinal cord transection with a graft; TA: tibialis anterior muscle; GM: gastrocnemius muscle; ECM: extensor caudae medialis muscle. Data normalized to the expression of *Htr7* gene in MNs of INT rats are presented as mean **±** SD (standard deviation). Nonparametric Kruskal-Wallis test with Conover post hoc method for multiple comparison (^∗^*p* < 0.05; ^∗∗^*p* < 0.01; ^∗∗∗^*p* < 0.001).

In SCI_1m_ rats, the *Htr7* gene expression was reduced by ~22% in TA MNs (nonparametric Kruskal-Wallis test (df: 3, *p* = 0.015) with Conover post hoc test *p* < 0.001), by ~50% in GM MNs (nonparametric Kruskal-Wallis test (df: 3, *p* = 0.022) with Conover post hoc test *p* < 0.05), and by ~20% in ECM MNs (nonparametric Kruskal-Wallis test (df: 3, *p* = 0.025) with Conover post hoc test *p* < 0.001) when normalized to the *Htr7* gene expression in MNs of INT animals (Figure 6).

In SCI_4m_ rats, the *Htr7* gene expression was also lower than in INT rats in TA MNs (Conover post hoc test *p* < 0.05) and in GM MNs (Conover post hoc test *p* < 0.01). There was also a small increase in gene expression in SCI_4m_ rats in comparison to SCI_1m_ rats (Conover post hoc test *p* < 0.05) in TA MNs but remained significantly lower in comparison to that of INT (*p* < 0.01). In MNs of GM muscle, *Htr7* gene expression was unchanged (Conover post hoc test *p* > 0.05) in comparison to that of SCI_1m_ rats but remained lower in comparison to that of INT (Conover post hoc test *p* < 0.01). In SCI_4m_, the *Htr7* gene expression in the ECM MNs increased by ~12% (Conover post hoc test *p* < 0.05) in comparison to the gene expression level measured in SCI_1m_ rats (Figure 6).

Intraspinal grafting of embryonic brainstem serotonergic neurons reversed changes in the *Htr7* gene expression observed in the TA and GM MNs of SCI_1m_ rats (Figure 6). Expression of the *Htr7* gene in the TA MNs (nonparametric Kruskal-Wallis test (df: 3, *p* = 0.015) with post hoc Conover method for multiple comparisons *p* < 0.001) and in the GM MNs (nonparametric Kruskal-Wallis test *p* = 0.022 and Conover post hoc adjusted with FDR method for multiple comparisons *p* < 0.05) in SCI_TR_ rats was higher than that in the INT rats. Expression of the *Htr7* gene in SCI_TR_ significantly increased by ~109% (Conover post hoc *p* < 0.01) and ~215% (Conover post hoc test *p* < 0.001) in the TA and GM MNs, respectively, in comparison to the *Htr7* gene expression in the SCI_1m_ and by ~86% (Conover post hoc *p* < 0.001) and ~215% (Conover post hoc test *p* < 0.001) in the TA and GM MNs, respectively, in comparison to the *Htr7* gene expression in SCI_4m_ rats. On the other hand, in the ECM MNs, expression of the *Htr7* gene in the SCI_TR_ rats was not different in comparison to the *Htr7* expression in the SCI_4m_ rats (nonparametric Kruskal-Wallis (df: 3, *p* = 0.025 with Conover post hoc *p* = 0.81)).

## 4. Discussion

In our previous papers, we demonstrated that intraspinal grafting of embryonic neurons destined to form the descending 5-HT system of the rat brain stem effectively restores coordinated plantar stepping in adult spinal rats [10, 13, 51, 57]. We also demonstrated that such recovery is mediated by 5-HT_2A_ and 5-HT_7_ serotonergic receptors [10, 13].

In the present paper, we confirmed expression of the *Htr2a* and *Htr7* genes in MN populations of intact and paraplegic rats. We showed for the first time that intraspinal transplantation of rat E14 embryo brainstem containing serotonergic neurons increases expression of these genes in the TA and GM hindlimb MNs in comparison to ungrafted spinal animals. We found that 5-HT_7_ receptor mRNA was decreased at 1 month and 4 months after spinal cord injury in all 3 types of MNs. In the presence of the grafts, 5-HT_7_ mRNA levels increased above those at 1 and 4 months and were significantly different from the intact condition in TA, GM, and ECM MNs. The graft tended to reverse the decrease in 5-HT_7_ receptor mRNA in TA and GM MNs, with a sustained increase after 4 months in the grafted rats. The grafts did not have such an effect in ECM (tail) MNs. One month after SCI, 5-HT_2A_ receptor mRNA decreased in flexor and extensor MNs, while it increased in tail MNs. Flexor and extensor MNs had decreased 5-HT_2A_ mRNA after 4 months, while 5-HT_2A_ mRNA in the tail MNs decreased in comparison to that observed one month after SCI but remained slightly higher than that in INT. Grafting resulted in 5-HT_2A_ mRNA levels that did not differ from the intact condition in GM MNs, but 5-HT_2A_ mRNA expression continued to be slightly downregulated in TA MNs and upregulated in ECM MNs. It is possible to conclude that the presence of the grafts tended to normalize the levels of 5-HT receptor mRNA in limb muscle MNs, as predicted by our hypothesis, but not in MNs of tail muscle. A possible mechanism for the effect of the grafts might be that increased *Htr2a* gene expression may be a consequence of the increased presence of 5-HT derived from the graft, which can occur in some cells [62]. The mechanism for regulation of *Htr2a* gene expression in MNs of different types may vary. This is a suitable topic for further research.

Our data showing 5-HT_2A_ mRNA upregulation in ECM is similar to findings on receptor protein upregulation in more distal tail muscle MNs [63–65]. In MNs innervating hindlimb muscles, 5-HT_2A_ receptor protein levels have been observed to be upregulated 4–6 weeks after contusive SCI [66, 67]. Protein levels for this receptor have not been examined at later time points after injury. Absence of an increase in *Htr2a* gene expression after total transection observed here is consistent with in situ hybridization data obtained by Ung and colleagues showing a clear tendency of 5-HT_2A_ mRNA to decrease in the ventral horn of lumbar segments 28 days after spinal cord transection in adult mice [68]. Our data on 5-HT_2A_ receptor mRNA are not predictive of the increase in 5-HT_2A_ receptor protein. Maier et al. [69] reviewed the common observation that mRNA and protein expression can be uncorrelated. They conclude that the major posttranslational factor influencing mRNA-protein correlation is the individual half-life of proteins. Our observation of a decrease in mRNA expression at times when increases in protein expression occur [66, 67] can be explained by the relative half-life of the mRNA and the 5-HT_2A_ receptor protein. In situ hybridization results [68] are consistent with a decrease in *Htr2a* gene expression after spinal cord injury in the ventral horn (28 days in their case and one month and 4 months in our case). In the paper by Chopek et al. [42], the samples were taken 3 months after injury. Since mRNA expression is not maintained in a steady state [69], some fluctuation in mRNA expression over time is to be expected. For example, in laser-captured phrenic motoneurons [70], mRNA for 5-HT_2A_ receptors was upregulated 14 days after cervical hemisection, but had returned to normal or below at 21 days. Fuller et al. [27], in contrast, found upregulation of 5-HT_2A_ protein after 7–14 days in the same preparation (cervical hemisection). The decrease in *Htr2a* gene expression may be an autoregulatory response to increased receptor density [71].

Chopek and others [42] found significant increases in *Htr2a* gene expression in motoneurons innervating hindlimb flexor and extensor muscles after spinal cord transection. This is not surprising given the frequent absence of correlation between gene and protein expression after injury. What might account for the differences between our findings and those of Chopek et al? They might be an effect of different selection of motoneurons for analysis. We compared MNs innervating TA with those innervating GM based upon the fact that these muscles have comparable composition of the three types of motor units, but they are different in function (extensor versus flexor). Chopek and others collected MNs innervating both soleus and gastrocnemius lateralis muscles to the extensor pool and MNs innervating extensor digitorum longus and tibialis anterior muscles to the flexor pool. Soleus is a typical slow muscle, and extensor digitorum longus is a typical fast muscle, so including these MNs in their extensor and flexor pools, respectively, may have influenced the results. MNs of different sizes may express 5-HT_2A_ receptors differently, and they react differently to spinal cord injury [72]. Moreover, Chopek and others [42] use *SDHA* as the reference gene for qPCR analysis, which may have influenced the results, because expression of this gene may be altered by spinal cord injury. This is the case after disuse in microgravity, and it appears to induce changes in *SDHA* expression differently in motoneurons of different sizes [73, 74]. We selected the *Ppia* gene after Navarrett and others [75], who verified it as suitable for use in spinal cord tissue after injury. We also eliminated any effects of 5-HT receptors in blood [76] by perfusing the rat with PBS before dissecting the spinal cord and further preparation for laser capture of the motoneurons, whereas this was not done by others who observed an increase in 5-HT_2A_ mRNA [42, 43]. In the Di Narzo et al. [43] study, the sample was taken from the sacral cord and was not limited to motoneurons. Another possible explanation for the differences between our observations on 5-HT_2A_ mRNA levels after injury and those of Chopek et al. [42] and Di Narzo et al. [43] is that they used Sprague-Dawley rats, while WAG (Wistar Albino Glaxo) rats were used in our study.

Di Narzo et al. [43] described recently that in rat spinal cord, the mRNA expression of *Htr2c* gene is influenced by inflammation caused by total transection at the sacral level (0.6 mm below and above the SCI). Our analysis of the inflammatory responses in the spinal cord of spinal rats with and without a graft revealed that in the ventral horn of the lumbar segments (6 mm below the graft site—the shortest distance below the graft that MNs of TA and GM muscles were collected), there was no increased level of astrocytes and no presence of activated microglia. Thus, inflammation induced by intraspinal grafting is not likely to be responsible for the observed changes in *Htr2a* and *Htr7* gene expression.

There is good evidence for constitutive activity in 5-HT_2_ receptors in sacral MNs after S2 spinal cord injury (reviewed in [14]), but the evidence that this is true in MNs innervating limb muscles is less complete. The degree of constitutive activity of 5-HT_2A_ receptors is thought to be dependent upon mRNA expression [75]. The reduction in mRNA after SCI may be interpreted as an indication of decreased constitutive activity in both 5-HT_2A_ and 5-HT_7_ receptors. Our finding that limb motoneuron 5-HT_2A_ and 5-HT_7_ mRNA expression is increased by the grafts could therefore reflect an increase in constitutive activity in these receptors. Whether this can account for the efficacy of the grafts for improving locomotor recovery in paraplegic rats will require further investigation. These results suggest that the mechanisms regulating 5-HT receptor mRNA expression in limb and tail MNs after SCI might be different.

Our results showing 5-HT_7_ receptor mRNA upregulation after grafting provides support for the suggestion that the graft improves locomotion by increasing the role of this receptor in these MNs. Both 5-HT_2A_ and 5-HT_7_ receptors are expressed in phrenic MNs, and both receptors facilitate spinal motor plasticity [77, 78]. Their coexistence results in mutual crosstalk inhibition (Fuller and Mitchell respiratory neuroplasticity) due to the downstream effects induced by Gq (5-HT_2A_) and Gs (5-HT_7_) activation. The details of this crosstalk inhibition are not well defined, but this factor needs to be taken into account if the function of neurons expressing both these G protein receptors is to be understood. In the case of limb motoneurons, it is possible that such crosstalk inhibition could result in mutual downregulation of receptor expression. Further investigations of the effects of 5-HT_7_ receptors on limb motoneurons are warranted. A description of the mechanisms involved in the increased expression of RNAs for both receptors induced by the grafts will require further investigation.

5-HT_7_ receptors appear to be expressed in limb MNs, but their role in controlling motoneuron function has not been established. Zhang [79] found that a 5-HT_7_ receptor agonist produced depolarization when applied iontophoretically onto lumbar MNs in cat spinal cord. MN excitability might be increased if 5-HT_7_ receptors mediated a reduction in the afterhyperpolarization in limb MNs, which appears to be the case in trigeminal MNs [19]. Limb MN AHPs are reduced by iontophoretically applied 5-HT [80], but the receptor responsible for this effect has not been determined. These issues need to be investigated, along with the possibility of inhibitory crosstalk between 5-HT_2A_ and 5-HT_7_ receptors which occurs in phrenic motoneurons [77, 78], when both receptors are present in the same cell.

## 5. Conclusion

Our results indicate that thoracic spinal cord transection leads to changes in *Htr2a* and *Htr7* gene expression, whereas transplantation of embryonic serotonergic neurons reverses these changes in MNs innervating hindlimb muscles but not those innervating the tail muscle. These differences in *Htr2a* and *Htr7* gene expression between MNs innervating hindlimb and tail muscles can be a result of the distance from the graft to the motoneurons. The fibers of grafted 5-HT neurons do not extend farther than to the level of L4-L5 lumbar segments [10, 57], so motoneurons innervating the ECM muscle may not be supplied by serotonergic innervation of graft origin. Alternatively, it may be that motoneurons that innervate tail muscles have unique adaptations to spinal injury and the effects of intraspinal grafts of 5-HT neurons, such as reliance on constitutive activity of 5-HT_2_ receptors after spinal cord injury. Increased *Htr2a* and *Htr7* gene expression induced by the graft could restore motoneuron excitability and lead to improved motor function, such as we observed in the grafted rats [10, 57]. Here, we show for the first time that changes in gene expression induced by the graft may account for this recovery.

## Figures and Tables

**Table 1:** Description of assays used in real-time PCR.

Gene symbol	Reference number	Gene name	Assay ID	Amplicon length
*Ppia*	NM_017101.1	Cyclophilin A	Rn00690933_m1	149
*Htr2a*	NM_017254.1	Receptor 5-HT_2A_	Rn00568473_m1	71
*Htr7*	NM_022938.2	Receptor 5-HT_7_	Rn00576048_m1	85

## Data Availability

All data used in this study are included in the article. Any additional information required will be provided upon request to the corresponding author.
